# Contrasting mitochondrial diversity of European starlings (*Sturnus vulgaris*) across three invasive continental distributions

**DOI:** 10.1002/ece3.6679

**Published:** 2020-08-27

**Authors:** Louise Hart Bodt, Lee Ann Rollins, Julia M. Zichello

**Affiliations:** ^1^ Educational Laboratory for Comparative Genomics and Human Origins American Museum of Natural History New York NY USA; ^2^ Cold Spring Harbor Laboratory DNA Learning Center Cold Spring Harbor NY USA; ^3^ Department of Biology New York University New York NY USA; ^4^ Evolution & Ecology Research Centre School of Biological, Earth and Environmental Sciences UNSW Sydney Sydney NSW Australia; ^5^ Division of Anthropology American Museum of Natural History New York NY USA; ^6^ Division of Natural Sciences College of Mt. St. Vincent Bronx NY USA

**Keywords:** avian genetics, European starlings, invasion genetics, invasive species, molecular evolution

## Abstract

European starlings (*Sturnus vulgaris*) represent one of the most widespread and problematic avian invasive species in the world. Understanding their unique population history and current population dynamics can contribute to conservation efforts and clarify evolutionary processes over short timescales. European starlings were introduced to Central Park, New York in 1890, and from a founding group of about 100 birds, they have expanded across North America with a current population of approximately 200 million. There were also multiple introductions in Australia in the mid‐19th century and at least one introduction in South Africa in the late 19th century. Independent introductions on these three continents provide a robust system to investigate invasion genetics. In this study, we compare mitochondrial diversity in European starlings from North America, Australia, and South Africa, and a portion of the native range in the United Kingdom. Of the three invasive ranges, the North American population shows the highest haplotype diversity and evidence of both sudden demographic and spatial expansion. Comparatively, the Australian population shows the lowest haplotype diversity, but also shows evidence for sudden demographic and spatial expansion. South Africa is intermediate to the other invasive populations in genetic diversity but does not show evidence of demographic expansion. In previous studies, population genetic structure was found in Australia, but not in South Africa. Here we find no evidence of population structure in North America. Although all invasive populations share haplotypes with the native range, only one haplotype is shared between invasive populations. This suggests these three invasive populations represent independent subsamples of the native range. The structure of the haplotype network implies that the native‐range sampling does not comprehensively characterize the genetic diversity there. This study represents the most geographically widespread analysis of European starling population genetics to date.

## Graphical abstract

European starlings represent one of the most widespread and problematic invasive species in the world. Therefore, understanding their population history and current population dynamics can contribute to conservation efforts and clarify recent evolutionary processes.Here, we compare mitochondrial diversity in birds from North America, Australia, South Africa, and the native range in the United Kingdom. This study represents the most geographically widespread analysis of European starling population genetics to date.

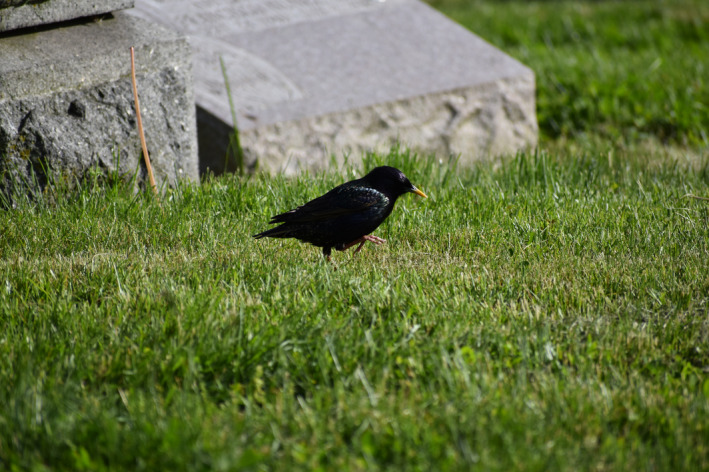

## 1 INTRODUCTION

Invasive populations are useful systems to investigate responses to novel environments, providing insight into mechanisms underlying invasion success and native species’ capacity to adapt to a changing world (Moran & Alexander, 2014). Despite this opportunity, these studies often examine only one introduction, reducing their power to draw robust conclusions that are broadly applicable (Packer et al., 2017). For this reason, there is a growing interest in studying invasive species that have been introduced to multiple geographically and environmentally diverse localities (Kueffer, Pyšek, & Richardson, 2013; Packer et al., 2017). In this respect, the European starling (*Sturnus vulgaris*) is an excellent system to investigate evolutionary responses to a wide range of introduced environments, from tropical Fiji to temperate Argentina (Pinto, 2005).

European starlings are native to the Palearctic but have been repeatedly introduced to novel environments, flourishing in their invasive ranges (Long, 1981). Starlings have now been introduced to every continent barring Antarctica (Rollins, Woolnough, & Sherwin, 2006, Figure 1). Their invasion success likely results from a suite of life‐history and behavioral traits that may facilitate ecological flexibility. For example, they are often classified as diet generalists, preferring insects, but they will eat most other foods depending on availability of resources (Cabe, 1993). Another feature that likely plays a role in European starlings’ ability to persist in new localities is their flexibility in patterns of seasonal migration. Although not all starling populations are migratory (e.g., in Australia and New Zealand, Higgins, Peter, & Cowling, 2006), in populations that are migratory, there is a great deal of individual variation in migratory behavior (i.e., individuals can be differentially migratory from year to year; Blem, 1981; Feare, 1984). Some research suggests that seasonal migration may be an adaptive strategy in response to seasonality; therefore, migratory flexibility in starlings may allow them to persist in seasonal environments and facilitate range expansion (Winger, Auteri, Pegan, & Weeks, 2019). This trait may also contribute to differences in population structure across introductions.

**Figure 1:**
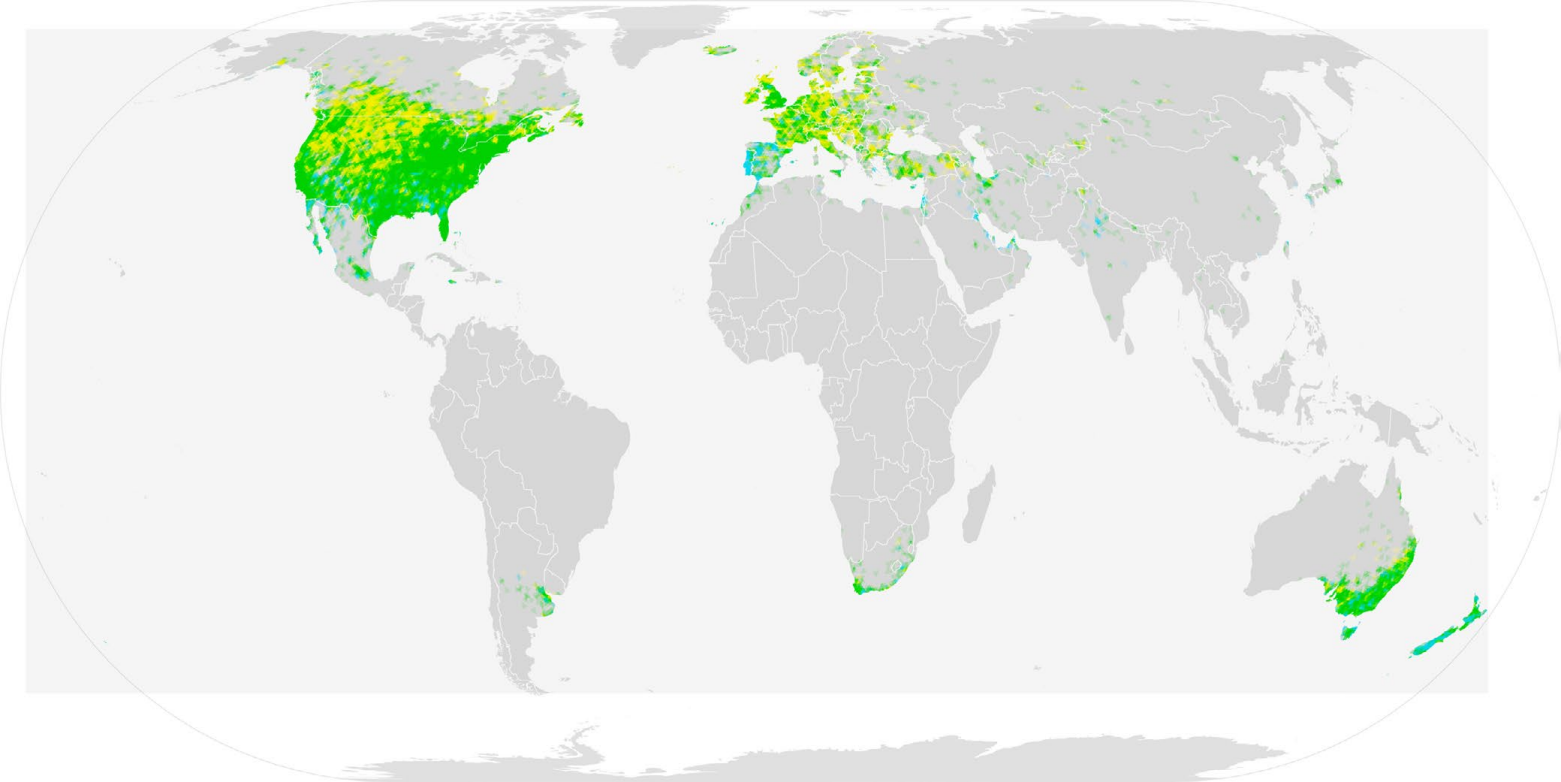
Map of worldwide distribution of starlings. Green = year‐round resident, yellow = summer resident, blue = winter resident. Source: Wikimedia commons https://commons.wikimedia.org/wiki/File:European_Starling_Range.png#file, generated from eBird Basic Dataset 2015

European starlings were introduced to North America in 1890 as part of an American Acclimatization Society initiative to populate Central Park with the birds from Shakespeare’s plays (Cooke, 1928; Phillips, 1928). The initial introduction consisted of approximately 60 individuals released in 1890 and 40 more in 1891, leading to a total of ~100 individuals released into Central Park in New York City (Cabe, 1993). From this founding population, starlings have expanded their range across all of North America where their current population exceeds 200 million individuals, over one‐third of the global population of this species (Feare, 1984). This range expansion has taken place in the last 130 years, demonstrating their ability to persist in a heterogeneous novel environment. Given the diverse environments colonized by starlings in North America, it is interesting that nuclear markers indicate that little population structure exists (allozymes, Cabe, 1998; single nucleotide polymorphisms, Hofmeister, Werner, & Lovette, 2019).

Other starling introductions from the 19th century have been previously studied, including the mid‐19th century Australian introductions (Rollins et al., 2016; Rollins, Woolnough, Sinclair, Mooney, & Sherwin, 2011; Rollins, Woolnough, Wilton, Sinclair, & Sherwin, 2009) and the late 19th century South African introduction (Berthouly‐Salazar et al., 2013). In Australia, up to sixteen different introduction attempts have been made with birds originating from the United Kingdom, from 1856 to 1881, with only two resulting in recorded established populations from ~165 original birds (Higgins et al., 2006; Long, 1981). Nuclear and mitochondrial markers identified concurrent population structure across the Australian range, and nuclear polymorphisms were associated with environmental variables in that population (e.g., aridity; Cardilini et al., 2020; Rollins et al., 2009, 2011). In contrast to the high levels of propagule pressure in Australia, only one introduction to South Africa of ~18 birds originating from Britain in or around 1897 has been recorded (Winterbottom & Liversidge, 1954). The South African introduction enables a powerful comparison with the North American introduction because of similarities in timing of these events (1897 and 1890, respectively). Both the Australian and South African introductions have reduced mitochondrial genetic diversity in comparison to the native source population in the UK (Berthouly‐Salazar et al., 2013; Rollins et al., 2011).

Founding population sizes during introduction are often small, resulting in genetic bottlenecks and lower genetic diversity than in the native range (Baker & Stebbins, 1965; Nei, Maruyama, & Chakraborty, 1975). However, numerous insights from studies of other invasions suggest that decreased genetic diversity at introduction may not hinder these species’ ability to become established in novel environments (Dlugosch, Anderson, Braasch, Cang, & Gillette, 2015; Frankham, 2005). Factors such as the number of introduction attempts, the timing of these attempts, dispersal patterns in the introduced range, and the rate of population expansion may play a larger role in shaping patterns of genetic diversity and ultimately contributing to successful colonization. A wide body of evidence suggests that adaptation in introduced ranges occurs rapidly, and this does not appear to be reliant on genetic diversity (Rollins et al., 2013).

Here, we use mitochondrial control region sequence data to examine starling population structure in North America and compare mitochondrial genetic diversity in populations from the native‐range and from three established invasions: North America, Australia, and South Africa. Although the limitations of using mitochondrial DNA in population genetic analyses have been well characterized (Ballard & Whitlock, 2004; Bazin, Glémin, & Galtier, 2006), there are several benefits associated with its use. First, previous studies of starlings in Australia, South Africa, and the UK used mitochondrial control region sequence data, so the comparative strength of our study is predicated on using the same marker. Second, Australian studies that have compared population structure using mitochondrial sequence data to that of microsatellite (Rollins et al., 2011) and single nucleotide polymorphism data (Cardilini et al., 2020) found similar patterns, supporting the validity of our approach. Third, mitochondrial DNA is still one of the most reliable sources of DNA that can be extracted from historical museum specimens (Guschanski et al., 2013; Mason, Li, Helgen, & Murphy, 2011; Ramakrishnan & Hadly, 2009), and population analyses using historical specimens rely on comparable datasets from modern birds, such as this. Finally, although mitochondrial DNA cannot provide a complete evolutionary picture, it is especially useful as evidence to clarify recent changes in a population (Zink & Barrowclough, 2008). This is especially true of the noncoding control region, which has high nucleotide diversity (Saccone, Pesole, & Sbisà, 1991).

In this study, we use this unique biological system that features multiple, independent, and documented introductions to investigate how propagule pressure (e.g., the number of introductions), environmental factors, and the expansion rate in introduced ranges influence contemporary population structure and genetic diversity. Based on previous research using nuclear markers, we predict low levels of population structure within North America. We predict that the mitochondrial diversity of the North American population will be lower than that of Australia, where multiple introductions were made (Jenkins, 1959), and these occurred prior to and had a greater number of propagules than the New York introduction (Australian introductions started in 1854; Jenkins, 1959). Further, we predict similar levels of genetic diversity in South Africa and North America, due to similarities in timing of introductions and propagule pressure. We discuss microevolutionary changes that have occurred since the introduction of these populations across the world.

## 2 METHODS

### 2.1 Samples and DNA extraction

North American tissue samples (*N* = 95) were obtained from starlings culled by the United States Department of Agriculture Animal and Plant Health Inspection Service (USDA APHIS) between 2014 and 2018 at 14 localities across the United States (Table 1). Samples were shipped in ethanol and frozen at −20°C upon arrival. For fresh samples collected by the USDA, DNA extraction was performed using the Qiagen Blood and Tissue Kit. Extractions were performed at room temperature, with an overnight incubation at 56°C to completely lyse muscle tissue. Elution buffer was warmed to 56°C and incubated for 30 min before the final spin and elution. Extracted DNA samples were stored at −20°C. DNA was also extracted from native‐range samples (*N* = 2) from National Museums Scotland, Edinburgh.

**Table 1:** Summary of number of starling specimens analyzed from each locality

North America		Australia		South Africa		Native Range	
Locality	Number of Samples	Locality	Number of Samples	Locality	Number of Samples	Locality	Number of Samples
Westchester, NY	10	Mason Bay, WA	31	Western Cape	158	Monks Wood, UK	27
Queens, NY	10	Jerdacuttup, WA	42	Eastern Cape	51	UK	16
Albany, NY	13	Munglinup, WA	34	Northern Cape	4	Central North Sea	1
Eglin AFB, FL	13	Coomalbidgup, WA	32	Free State	6	Aberdeenshire, SCT	1
Brandon, NE	4	Condingup, WA	34				
Ogallala, NE	1	Condingup, WA	29				
Bruneau, ID	3	Nullarbor, SA	30				
Hammett, ID	2	Coorabie, SA	48				
San Angelo, TX	5	Streaky Bay, SA	32				
Auxvasse, MO	5	Tumby Bay, SA	30				
Fort Morgan, CO	5	Stansbury, SA	31				
Los Angeles, CA	15	Mallala, SA	36				
Burbank, WA	5	McLaren Vale, SA	41				
Juneau, AK	4	Meningie, SA	30				
		Yarra Valley, VIC	32				
		Orange, NSW	35				
		Devonport, TAS	29				
		Hobart, TAS	21				
Total	95	Total	597	Total	219	Total	45

Sequences from starlings sampled on other continents were downloaded from GenBank including those from the native range (*N* = 43; Berthouly‐Salazar et al., 2013; Rollins et al., 2011; GenBank KF638591–617; HQ263631–42), Australia (*N* = 597; Rollins et al., 2011; GenBank 178 FJ542126.1–FJ542131.1, FJ542133.1, HQ2636230–HQ263630), and South Africa (*N* = 219; Berthouly‐Salazar et al., 2013; GenBank KF638591–617). These samples were analyzed together with the North American samples (see below). The total number of individuals included in the study was 956 (Table 2).

**Table 2:** Summary statistics for starling mitochondrial control region sequence data (928 bp) from native and invasive range populations

	North America	Australia	South Africa	United Kingdom	Overall
Sample size	95	597	219	45	956
# of haplotypes	16	15	15	30	64
*π*	0.005 ± <0.001	0.005 ± <0.001	0.005 ± <0.001	0.007 ± <0.001	–
*h*	0.876 ± 0.001	0.703 ± 0.001	0.779 ± 0.001	0.972 ± 0.001	–
*R*	14.7	7.7	10.0	30.0	21.0
*D*	0.142 (*p* = .63)	2.56 (*p* = .99)	2.34 (*p* = .99)	−0.25 (*p* = .48)	1.20 (*p* = .77)
*F_s_*	−0.84 (*p* = .45)	3.94 (*p* = .87)	1.31 (*p* = .72)	−16.85 (*p* < .000)	−3.11 (*p* = .51)

Note

Sample size, number of haplotypes, pairwise nucleotide diversity (*π*), haplotype diversity (*h*), haplotype richness (*R*), Tajima’s (*D*), and Fu’s (*F_s_*) neutrality test values are given for each population. Variability estimates are standard error.

### 2.2 Amplification and sequencing

The primers used to amplify the mitochondrial control region in North American specimens were initially designed to analyze mitochondrial diversity of the Australian population (Rollins et al., 2011). Rollins et al. (2011) designed a series of overlapping primers to be utilized in the amplification of museum specimens or highly degraded samples (Table S1). We used these primers to sequence the control region of North American samples in four overlapping segments. Two of these primers (svCRL1 and svPheH3) amplify most of the mitochondrial control region and also were used to amplify DNA from the starling population in South Africa (Berthouly‐Salazar et al., 2013).

For the PCRs, *PuReTaq Ready‐To‐Go* PCR Beads were rehydrated with 13.5 µl of molecular grade water, 5 µl of 10 µM forward and reverse primers, and 1.5 µl of DNA. The thermocycling conditions used here were identical to those described in the original paper (Rollins et al., 2011). This included a 5‐min step at 94°C, 30 cycles of 94°C for 30 s, 53°C for 15 s and 72°C for 30 s and a final extension step for 10 min at 72°C. PCR products were sent to GENEWIZ, Inc. (South Plainfield, NJ) for PCR clean up via an enzymatic purification. Sequencing reactions were performed by GENEWIZ, Inc. using Applied Biosystems BigDye version 3.1 and forward primers. The reactions were then sequenced on an Applied Biosystems 3730xl DNA Analyzer.

### 2.3 Population and expansion analysis

Overlapping sequences were aligned using the software *Geneious* 11.1.2 (Kearse et al., 2012) to generate a consensus sequence for each individual from North America. All subsequent alignments including the samples from other continents were generated on *Geneious* using the standard settings and the *Geneious* alignment algorithm (Kearse et al., 2012). Median joining haplotype networks were created using *Network v10.1.0.0* (Bandelt, Forster, & Röhl, 1999) and postprocessed using the maximum parsimony calculation to remove unnecessary median vectors (Polzin & Daneshmand, 2003). Final networks were produced using *Network Publisher v2.1.2.5* (Fluxus Engineering, Clare, UK). Networks were constructed for the North American samples (1181 bp) and for the full dataset. To construct the latter, we trimmed the full dataset to 928 bp to accommodate the continental dataset with the shortest sequence length.

Using the 928 bp dataset, we calculated fixation indices (*F*_ST_ values), pairwise nucleotide diversity (*π*), haplotype diversity (*h*), Fu’s *F_s,_* and Tajima’s *D* in Arlequin v3.5.1.2 (Excoffier & Lischer, 2010). Haplotype richness (*R*) for each population was calculated using *FSTAT v.2.9.4* (Goudet, 2003). Mismatch analyses were also conducted in Arlequin, but with the full dataset from each invasive population (see Table S5).

## 3 RESULTS

**Figure 2:**
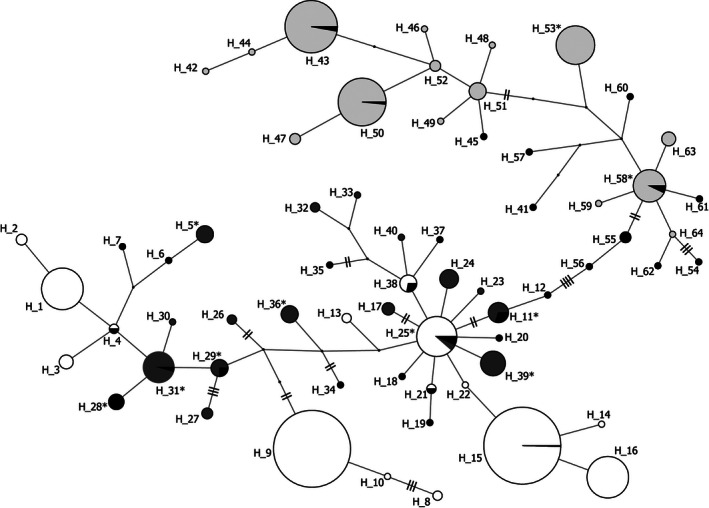
Median joining network of starlings from the native‐range (United Kingdom, black) and three invasive populations (North America, dark gray; Australia, white; South Africa, light gray) constructed using 928 bp of mitochondrial control region haplotypes. Median vectors are shown as small black dots. Distance between each node is equal to one mutation, except where noted by hash marks. Circle size indicates haplotype frequency. Asterisks denote haplotypes that contain polymorphisms in the 1181 bp dataset (see Table S4).

**Figure 3:**
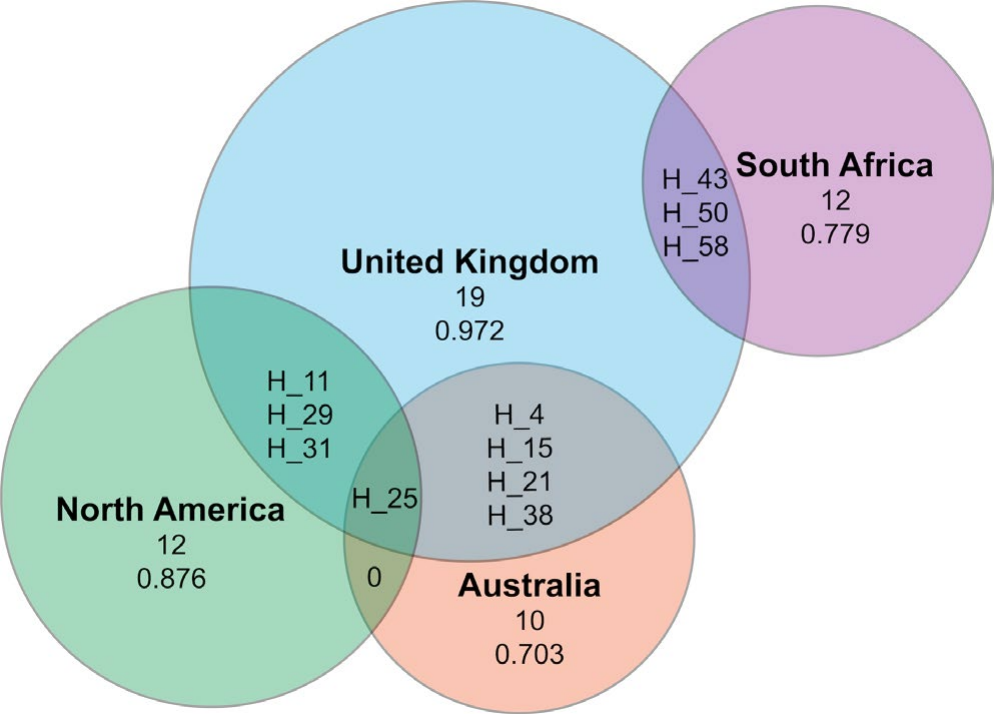
Venn diagram showing the number of haplotypes and the haplotype diversity for the native‐range population and the three invasive populations (North America, Australia, South Africa). The total number of unique haplotypes and haplotype diversity values are listed under the name of each locality. Shared haplotypes are listed by name within each intersection.

The haplotype network constructed using only the North American specimens (1,181 bp sequence) included 20 haplotypes encompassing 53 polymorphic sites and did not indicate the presence of regional population structure (Figure S1). When we included samples from all continents (928 bp sequence; Figure 2), we identified 64 haplotypes encompassing a total of 46 polymorphic sites (Table S5). Only one of these haplotypes was shared between two introduced regions (H_25; North American and Australia, Figures 2 and 3). Although haplotypes from North America and Australia were genetically similar, South African haplotypes were completely separated from the other invasive haplotypes by a minimum of eight mutations. Four native‐range haplotypes were found in North America (H_11, H_25, H_29, H_31). Overall, native‐range haplotypes were well distributed across the four‐continent network but not all basal haplotypes (e.g., H_51) were represented in the native‐range samples. Table S5 indicates polymorphic positions across haplotypes from all four populations and integrates naming conventions from the present study, and those of Australian and South African populations.

*F*_ST_ values from pairwise comparisons between the native‐range and the three introduced populations ranged between 0.060 (North America) and 0.174 (Australia) and were all statistically significant (Table S2). When the North American population was separated into three separate regions (Eastern, Central, and Western sampling sites), the *F*_ST_ values for all comparisons were low (*F*_ST_ ≤ 0.044) and only significantly different for the Central versus Western US comparison (Table S3).

**Figure 4:**
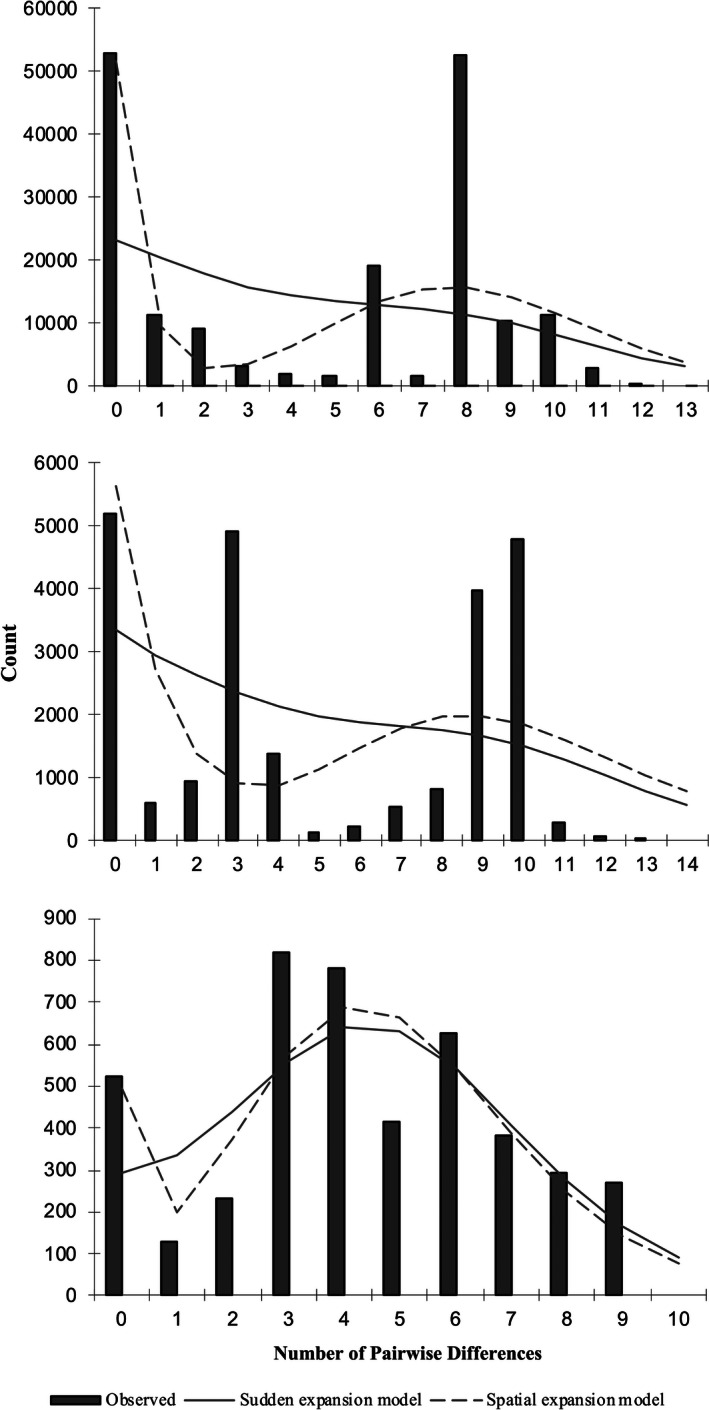
Mismatch distribution of the three invasive populations. Observed mismatches are represented by bars, and expected mismatches under each model are represented by lines (demographic expansion, solid line; spatial expansion, dotted line). Goodness‐of‐fit tests indicated that observed values were consistent with models in all cases except the demographic expansion model in South Africa

Haplotype diversity and richness were highest in the native range, followed by the North American population (Table 2). Tajima’s *D* values were nonsignificant in all four populations. Fu’s *F_s_* values were negative in North America and the native range, but only significant in the latter (Table 2). The mismatch distribution model for sudden (demographic) expansion was significantly different than empirical data from the South African population (SSD = 0.081, *p* = .04) but not from the Australian (SSD = 0.107, *p* = .07) nor North American populations (SSD = 0.15, *p* = .10) (Figure 4). The mismatch distribution model for spatial expansion was not different to that of empirical data from any of the three invasive populations (North America: SSD = 0.009, p = .56; Australia: SSD = 0.057, p = .26; South Africa: SSD = 0.075, *p* = .06; Figure 4).

## 4 DISCUSSION

Starlings are a highly successful invasive species occupying a wide breadth of environments across the world, resulting from introductions of varying age and intensity. This system enables a unique opportunity to study molecular evolution and adaptation. Here we use mitochondrial sequence data to compare the population genetic structure and diversity of the three best‐studied starling invasions: North America, Australia, and South Africa. Overall, our findings and those from data of other studies included here suggest that low genetic diversity is not an obstacle for this species’ rapid expansion and establishment in new environments (Dlugosch & Parker, 2008; Rollins et al., 2013).

As expected, the invasive populations had lower genetic diversity than the population in the native range, likely caused by genetic bottlenecks at introduction. The highest haplotype richness (which accounts for differences in sample size) was found in the UK (*R* = 30.0); although only 45 individuals were sampled, we identified 30 haplotypes in this population. Surprisingly, despite higher propagule pressure in Australia as compared to that of North America or South Africa, Australia harbored the lowest haplotype richness (*R* = 7.7). The North American population, which was intermediate in terms of propagule pressure, has retained the most genetic diversity (*R* = 14.7). Given the timescales involved, this is unlikely to be caused by novel mutations arising in North America (but see Rollins et al., 2016). However, it could be caused by differences in genetic diversity of founders or by higher levels of differential survival between haplotypes in Australian or South African starlings as compared to those from North America. It may be that some haplotypes have been lost in the native range since founders were collected. Differences in population expansion rates in novel environments also could be responsible for the differences in genetic diversity we found, with faster expansion resulting in higher haplotype diversity and lower nucleotide diversity (Halliburton & Halliburton, 2004).

The haplotype network including all populations (Figure 2) revealed some interesting relationships among haplotypes. South African starlings are genetically distinct from those of North America and Australia, suggesting that the founders for this population may have been sourced from a different region of the UK. North American and Australian starlings are genetically similar (intermixed in the network), but only shared a single haplotype (H_25), suggesting that the founders for these populations may have been sourced from the same region of the UK, but were likely to have been genetically distinct. As expected, UK samples were well distributed across the network, but many of the invasive haplotypes were not found in UK samples, highlighting the paucity of information that exists about starlings in their native range and making it difficult to further interpret sources of founding populations. For this reason, and because European starling populations are in decline in their native range (Heldbjerg et al., 2019), it may be important to further characterize this population.

Previous studies have investigated population structure within introduced populations of starlings. Within Australia, genetically distinct groups of starlings have been characterized using nuclear and mitochondrial markers (Rollins et al., 2009, 2011) and evidence of local adaptation to the Australian environment has been described (Cardilini, Buchanan, Sherman, Cassey, & Symonds, 2016; Cardilini et al., 2020). However, in South Africa, no evidence of population structure was found (Berthouly‐Salazar et al., 2013). The regional analysis conducted within North America in the present study also found little evidence of population structure in this invasive population. We did see a slight (*F*_ST_ = 0.04) albeit statistically significant difference between Central and Western samples but this may be due to the low sample size from the Central United States (*N* = 20). Overall, our findings are consistent with an earlier investigation of this population, which utilized allozyme data (Cabe, 1998), and a recent study using genome‐wide SNPs (Hofmeister et al., 2019). However, the latter indicated that there are genotypes associated with specific environmental features such as precipitation and/or temperature. This may imply that over time, population structure could develop in this invasive population, despite apparent high levels of dispersal. Interestingly, migration rates between Central and Western sites differ (Hofmeister et al., 2019) and banding data in North America have shown that the starlings are found to migrate in unpredictable ways, not always in the North and South direction, but also in the East and West directions (Brewer, 2000). Therefore, the genetic pattern we found may be due to the high dispersal rates and these unpredictable and latitudinal migration patterns.

When we investigated genetic differentiation across continents, we found that invasive populations were genetically divergent (*F*_ST_ ranged from 0.17 to 0.26, all statistically significant) and all significantly different from populations in the native range (*F*_ST_ ranged from 0.06 to 0.17). North America was most similar to the UK and Australia was least similar. These differences are likely caused by a combination of discrete introduction sources and founder effects. However, this could also be due to differences in timing of introductions; the Australian introduction occurred earlier than the others (mid‐19th century) so it is possible that these differences reflect shifts that occurred in the native range in the latter half of the 19th century.

Not surprisingly, we found genetic evidence of spatial expansion in all three invasive populations. While there was genetic support for demographic expansion in both North America and Australia, the mismatch analysis of South African data did not support the sudden (demographic) expansion model (Figure 4). This may mean that the South African starling population may still be in the “lag phase”, which typically occurs following introduction (Sakai et al., 2001). Neither Tajima’s *D* nor Fu’s *F_s_* values supported the presence of population expansion in any of the invasive populations. However, Fu’s *F*_s_ was significantly negative in the native range, which suggests that this population may either be undergoing expansion or that it has an excess of recent mutations (Fu, 1997). Given observations of population decline in the native range (described above), this might be a signal of directional selection, which could be a response to novel environmental stressors resulting from land use changes in the UK (Heldbjerg et al., 2019).

It is also interesting to consider that differences in the environments of each of the three invasive ranges studies here may have influenced population expansion rates. The United Kingdom and surrounding parts of Europe (native range) are largely classified as temperate with a hot or warm summer (Beck et al., 2018). Temperate areas similar to the native range are the regions where most starling invasive range expansion has occurred. The starling population in North America is about the same latitude as that of the native range between 40°–55°N, whereas the invasive populations in Australia and South Africa occur at about 30°–35°S (Sullivan et al., 2009). In Australia and South Africa, starlings have not expanded to cover the same area that they have in a comparable amount of time in North America. In North America, starlings spread from New York to Alaska from 1890 to 1970, which represents 80 years and a rate of 90 km/year (Bitton & Graham, 2014). In Australia, starlings rapidly expanded their range into south‐eastern Australia and were in Western Australia by the 1970s. However, starlings have not colonized the arid center (Higgins et al., 2006) of the continent, where the highest temperatures and lowest rainfall occur (Jones, Wang, & Fawcett, 2009). In South Africa, starlings spread primarily eastward from Cape Town, and are now reported only as far north as Kruger National Park (Berthouly‐Salazar et al., 2013; Sullivan et al., 2009). Similar to Australia, large areas of South Africa are classified as an arid, hot, desert with surrounding areas classified as arid, hot, steppe (Beck, 2018). These environmental differences pose an explanation for the rapid and continued expansion of European starlings in North America and associated mitigation of loss of genetic diversity, and suggests that the success of starlings in South Africa and Australia may have depended upon adaptation to novel climatic conditions.

There is still much to learn about the population dynamics and genetic structure of European starling invasions worldwide and about the native‐range genetics of this species. The mitochondrial dataset we have extended here is a useful tool to grow our knowledge of this species and, more generally, of invasion genetics. Despite our knowledge gaps, starlings provide an intriguing framework to study invasions of different ages and geographic extent (e.g., South America (small) versus Australia (large), of similar or contrasting genetic backgrounds (e.g., North America versus Australia (genetically similar), North America versus South Africa, (genetically different) and across different environments (e.g., North America (temperate) versus Fiji (tropical)). Together with the recent development of genomic resources (transcriptome: Richardson, Sherwin, & Rollins, 2017; genome (GCF_001447265.1: Nucleotide [Internet])), the features of this species make it ideal for advancing our knowledge of evolution in introduced ranges. Especially with continued global climate change, closely monitoring invasive species and understanding their outsized adaptive flexibility will be increasingly important to our ability to manage invasions and understand how species adapt to a changing world.

## CONFLICT OF INTEREST

None declared.

## AUTHOR CONTRIBUTIONS

**Louise Hart Bodt**: Formal analysis (lead); investigation (lead); writing–original draft (lead); writing–review and editing (equal). **Lee Ann Rollins**: Formal analysis (supporting); investigation (supporting); methodology (equal); supervision (supporting); writing–review and editing (equal). **Julia M. Zichello**: Conceptualization (lead); investigation (equal); supervision (lead); writing–review and editing (equal).

## Supporting information

Supplementary Material

## Data Availability

DNA sequences: GenBank accessions FJ542126.1–FJ542131.1, FJ542133.1, HQ2636230–HQ263630, KF638591–617. Novel DNA sequences: GenBank accessions: MT795633‐MT795650.
