# Supplementary material for: Contrasting mitochondrial diversity of European starlings (Sturnus vulgaris) across three invasive continental distributions
Source: Ecol Evol. 2020 Aug 27;10(18):10186–95. doi: 10.1002/ece3.6679 (PMC7520211; doi:10.1002/ece3.6679)

**Contrasting Mitochondrial Diversity of European Starlings (*Sturnus vulgaris*) Across Three Invasive Continental Distributions**

Authors: Louise Hart Bodt^1,2^, Lee Ann Rollins^3^, Julia Zichello^2,4^

**Supplementary Material**

**Table S1** Primers used were taken from primer set (Rollins et al., 2011).

| **Primer** | **Sequence** | |
| --- | --- | --- |
| svCRL1 | | 5’ - ACTTTTTCTCGTGCTTTAAGGGAT |
| svCRL2 | | 5’ - AGAGACATTCTTGTTTCAGGTAC |
| 4A/L437 | | 5’ - CTCACGAGAACCGAGCTACT |
| svCRL3 | | 5’ - GATAAGGTCGTCGCATACCC |
| svPheH3 | | 5’ - GCCGTCTTGACATCTTCAGT |

**Table S2** Global population differentiation. Lower matrix includes F_ST_ values generated using mtCR sequence data for three non-native populations (US, Australia and South Africa) and a native UK population of Sturnus vulgaris (asterisk indicates significance). Upper matrix contains *P* values.

|  | **United Kingdom** | **North America** | **Australia** | **South Africa** |
| --- | --- | --- | --- | --- |
|  |  |  |  |  |
| **United Kingdom** | --- | <0.001 | <0.001 | <0.001 |
| **North America** | 0.060* | --- | <0.001 | <0.001 |
| **Australia** | 0.174* | 0.221* | --- | <0.001 |
| **South Africa** | 0.133* | 0.171* | 0.263* | --- |

**Table S3** Regional population differentiation with the US. Lower matrix includes F_ST_ values generated from mtCR sequence data, investigating potential regional differences within North America (asterisk indicates significance). Upper matrix contains *P* values. Eastern US includes New York and Florida (N=46). Central US includes Missouri, Texas, Colorado and Nebraska (N=20). Western US includes Washington, Idaho, California and Alaska (N=29).

|  | **Eastern US** | **Central US** | **Western US** |
| --- | --- | --- | --- |
|  |  |  |  |
| **Eastern US** | --- | 0.171 ±0.032 | 0.126 ±0.031 |
| **Central US** | 0.018 | --- | 0.027 ±0.027 |
| **Western US** | 0.018 | 0.044* | --- |

| **Sample ID** | | **Locality** | **Sample ID** | **Locality** | **Sample ID** | **Locality** |
| --- | --- | --- | --- | --- | --- | --- |
| JMZ 1 | NY | | JMZ 34 | NY | CO03_1 | CO |
| JMZ 2 | NY | | JMZ 35 | NY | CO03_2 | CO |
| JMZ 3 | NY | | JMZ 36 | NY | CO03_3 | CO |
| JMZ 4 | NY | | JMZ 37 | NY | CO03_4 | CO |
| JMZ 5 | NY | | JMZ 38 | NY | CO03_5 | CO |
| JMZ 6 | NY | | JMZ 39 | NY | ID01_1 | ID |
| JMZ 7 | NY | | JMZ 40 | NY | ID01_2 | ID |
| JMZ 8 | NY | | JMZ 41 | NY | ID01_3 | ID |
| JMZ 9 | NY | | JMZ 42 | NY | ID03_1 | ID |
| JMZ 10 | NY | | JMZ 43 | NY | ID03_2 | ID |
| JMZ 11 | CA | | JMZ 44 | NY | MO2_1 | MO |
| JMZ 12 | CA | | JMZ 45 | NY | MO2_2 | MO |
| JMZ 13 | CA | | JMZ 46 | NY | MO2_3 | MO |
| JMZ 14 | CA | | JMZ 47 | NY | MO2_4 | MO |
| JMZ 15 | CA | | JMZ 48 | NY | MO2_5 | MO |
| JMZ 16 | CA | | JMZ 60 | FL | NE S1_1 | NE |
| JMZ 17 | CA | | JMZ 61 | FL | NE S1_2 | NE |
| JMZ 18 | CA | | JMZ 62 | FL | NE S1_3 | NE |
| JMZ 19 | CA | | JMZ 63 | FL | NE S1_4 | NE |
| JMZ 20 | CA | | JMZ 64 | FL | NE C2_1 | NE |
| JMZ 21 | CA | | JMZ 65 | FL | TX08_1 | TX |
| JMZ 22 | CA | | JMZ 66 | FL | TX08_2 | TX |
| JMZ 23 | CA | | JMZ 67 | FL | TX08_3 | TX |
| JMZ 24 | CA | | JMZ 68 | FL | TX08_4 | TX |
| JMZ 25 | CA | | JMZ 69 | FL | TX08_5 | TX |
| JMZ 26 | NY | | JMZ 70 | FL | WA01_1 | WA |
| JMZ 27 | NY | | JMZ 71 | FL | WA01_2 | WA |
| JMZ 28 | NY | | JMZ 72 | FL | WA01_3 | WA |
| JMZ 29 | NY | | NAB 91 | AK | WA01_4 | WA |
| JMZ 30 | NY | | NAB 92 | AK | WA01_5 | WA |
| JMZ 31 | NY | | NAB 94 | AK | UK 3 | Scotland |
| JMZ 32 | NY | | NAB 95 | AK | UK 4 | Scotland |
| JMZ 33 | NY | |  |  |  |  |

**Table S4** Sample ID for DNA samples extracted in this study, DNA located at the American Museum of Natural History.


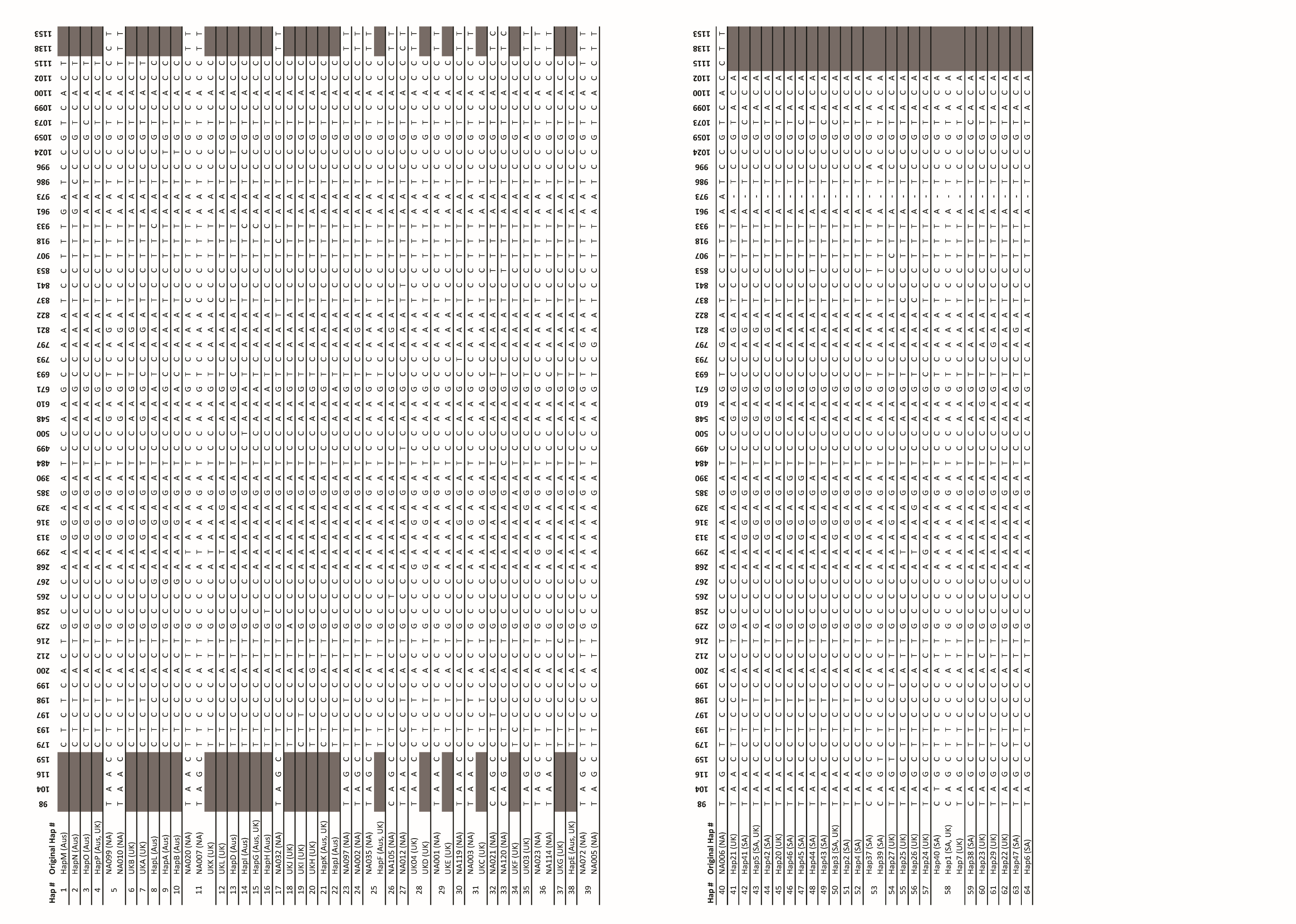


**Table S5** Polymorphic positions in starling mitochondrial control region sequence including individuals from the native range (United Kingdom, UK) and three invasive populations (North America, NA; Australia, Aus; and South Africa, SA). Column 1 contains haplotypes described in Figure 2. Column 2 indicates the equivalent haplotype name used in analyses of individual invasive populations. Note that some haplotypes contained in Figure 2 have polymorphisms outside of the 928bp sequence used in that analysis. Shading indicates missing data.


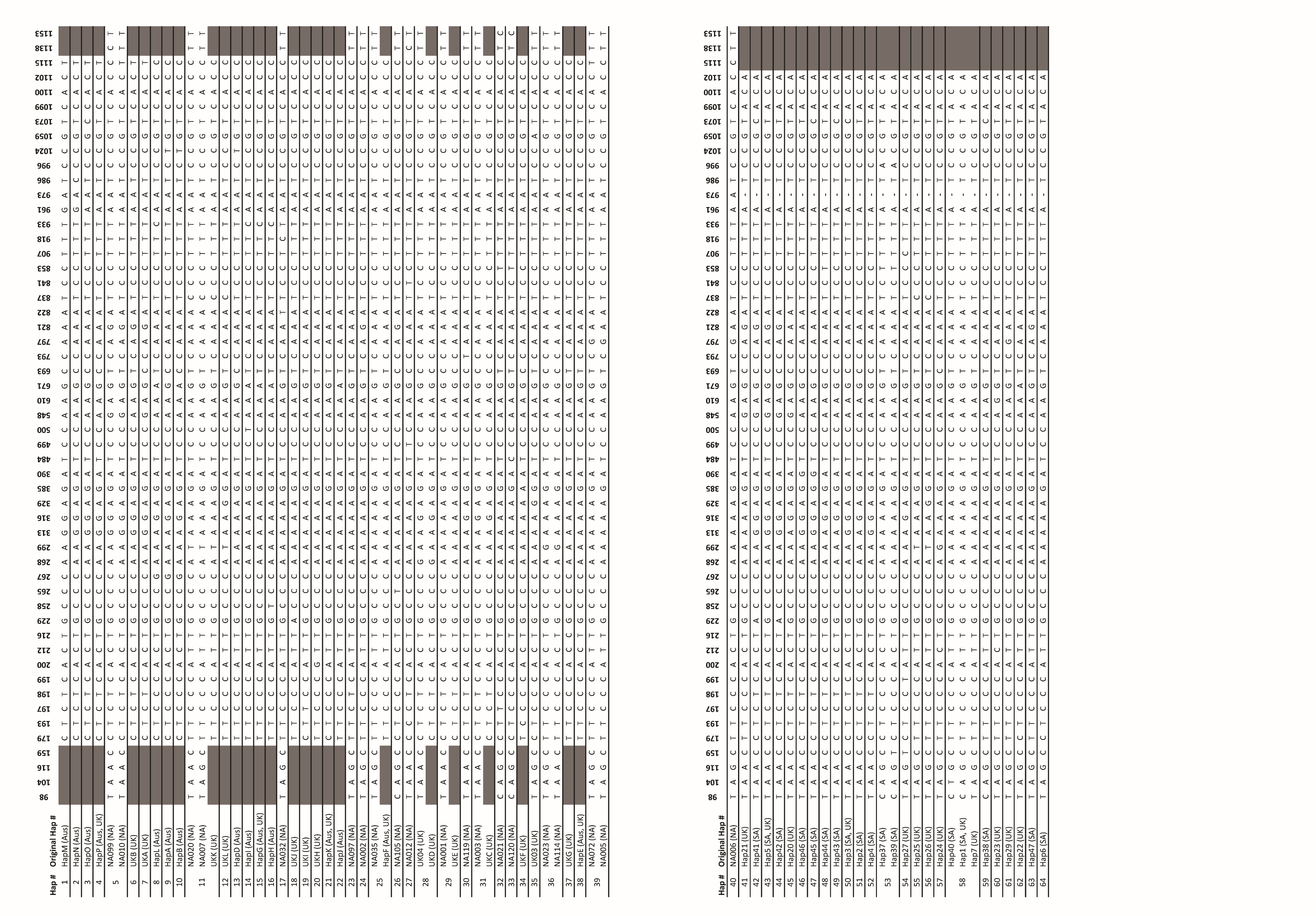


**Table S6** Haplotype summary. Table contains all haplotypes described here and in previous studies including haplotype number (used in Figure 2), total frequency, frequency by population (continent), name used in individual invasion studies and continent name.


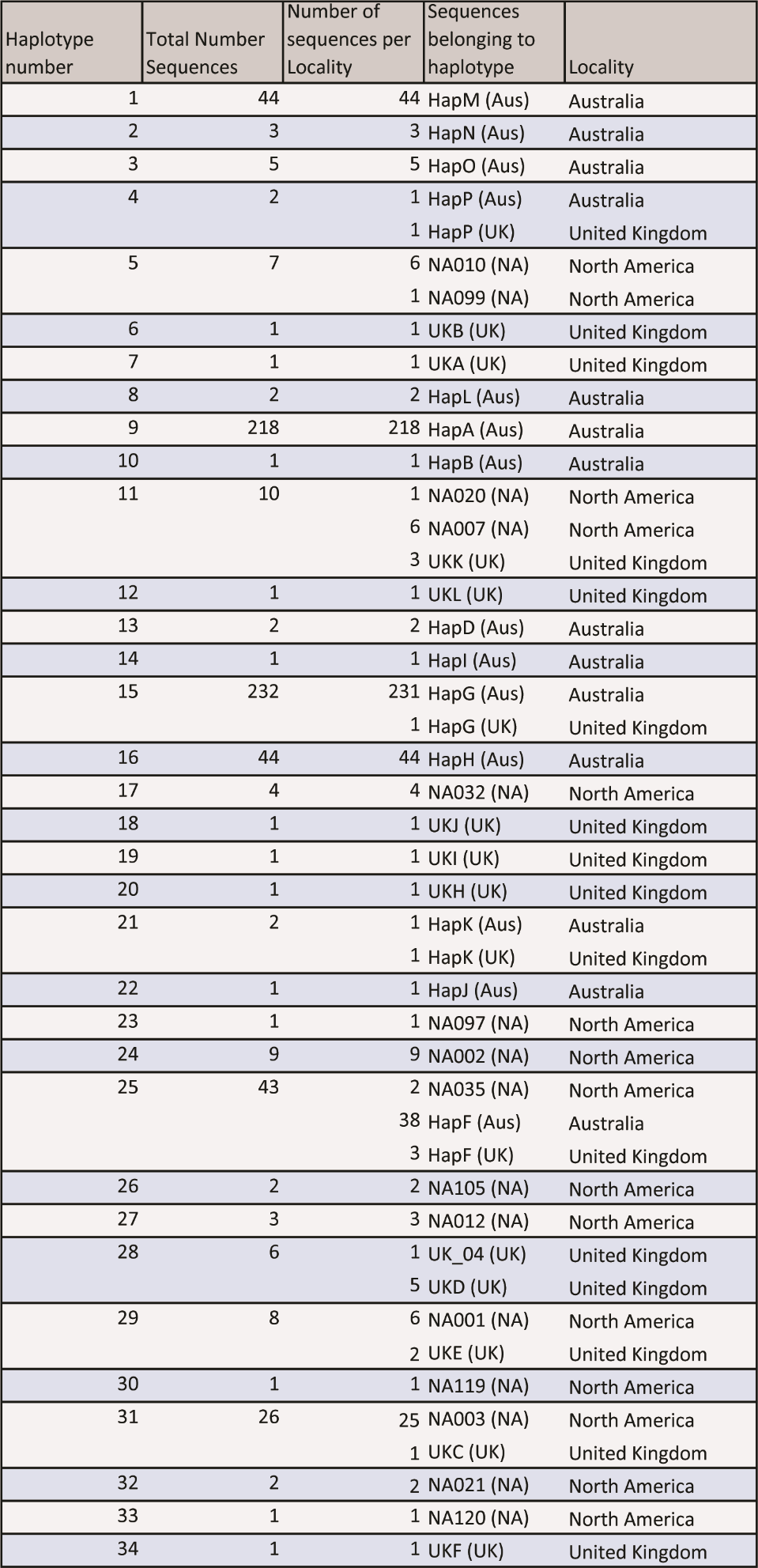


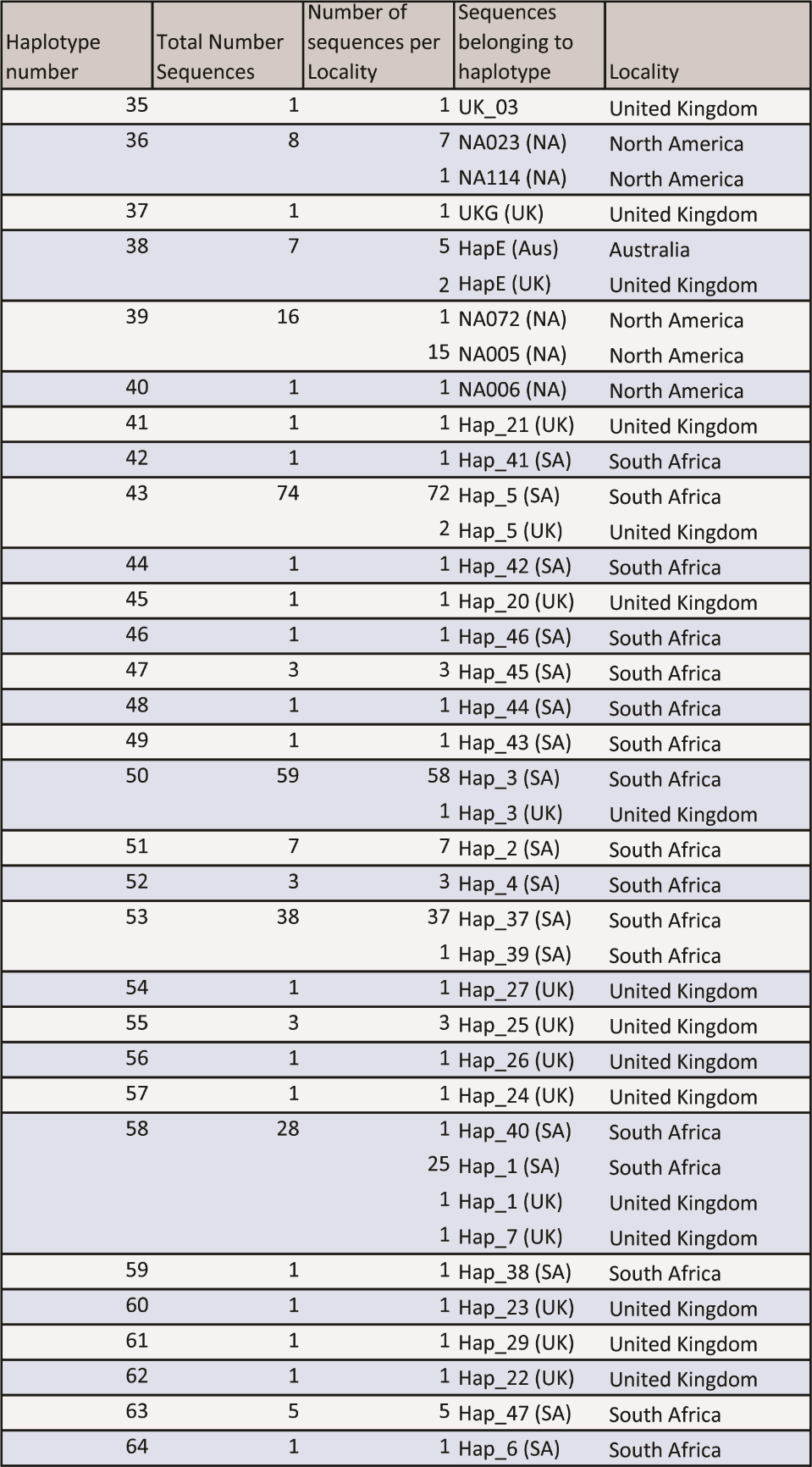


**Figure S1** Median joining network of North American starling mitochondrial control region haplotypes (1181bp). Haplotypes are categorized by region (Eastern US, black; Central US, gray; Western US, white). Median vectors are shown as small black dots. Distance between each node is equal to one mutation, except where noted by hashmarks. Circle size indicates frequency of haplotype.


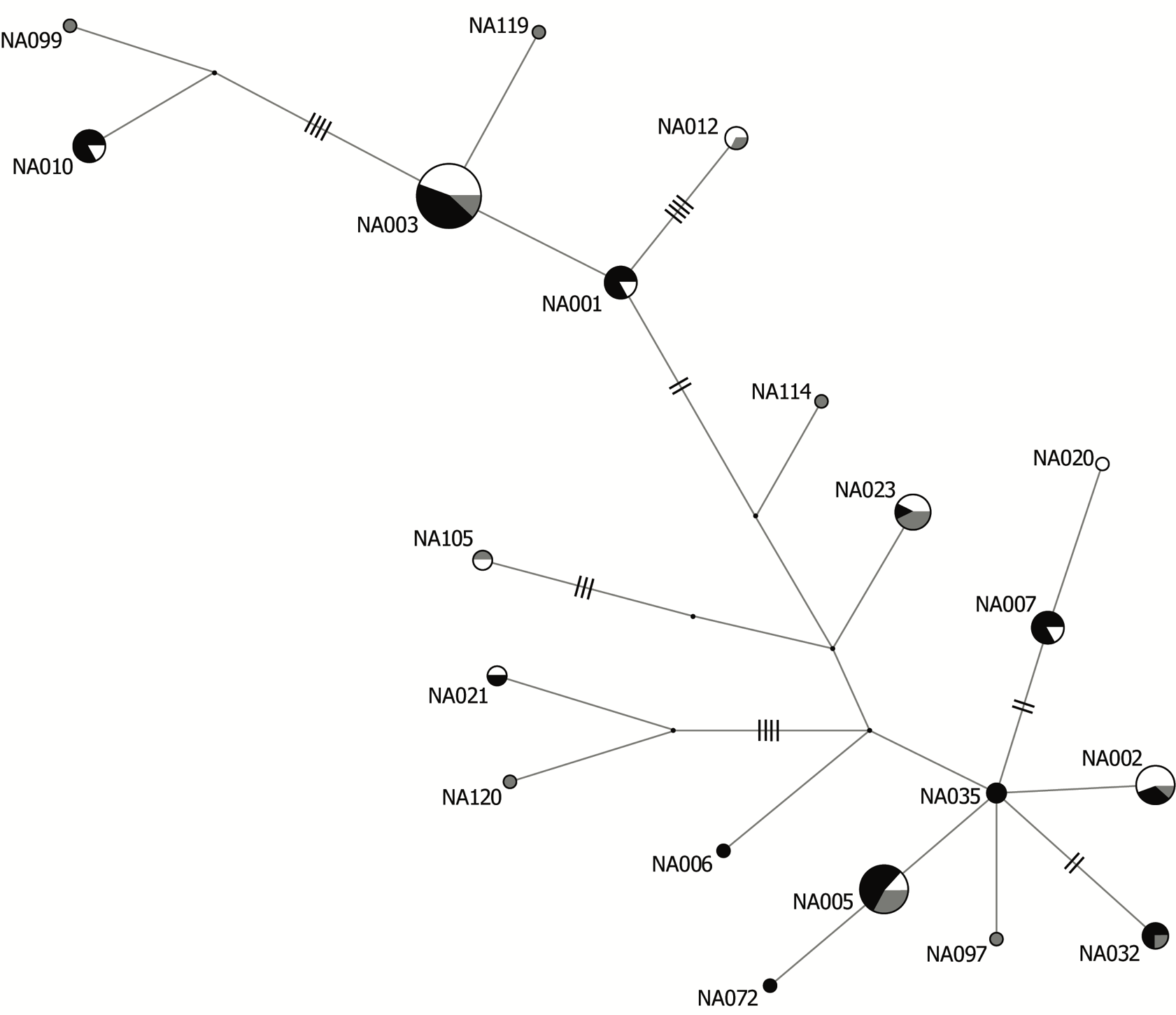

Supplement: Supplementary file 1 — Supplementary Material [file ECE3-10-10186-s001.docx]
